# Comparison of breast cancer and cervical cancer stage distributions in ten newly independent states of the former Soviet Union: a population-based study

**DOI:** 10.1016/S1470-2045(20)30674-4

**Published:** 2021-03

**Authors:** Anton Ryzhov, Marilys Corbex, Marion Piñeros, Anton Barchuk, Diana Andreasyan, Sayde Djanklich, Vadim Ghervas, Olga Gretsova, Dilyara Kaidarova, Konstantin Kazanjan, Fuad Mardanli, Yuriy Michailovich, Elena Ten, Alesya Yaumenenka, Freddie Bray, Ariana Znaor

**Affiliations:** a Cancer Surveillance Branch, International Agency for Research on Cancer, Lyon, France; b National Cancer Registry of Ukraine, National Cancer Institute, Kyiv, Ukraine; c Taras Shevchenko National University of Kyiv, Kyiv, Ukraine; d WHO Regional Office for Europe, Copenhagen, Denmark; e Tampere University, Faculty of Social Sciences/Health Sciences, Tampere, Finland; f NN Petrov National Research Medical Center of Oncology, Saint Petersburg, Russia; g National Institute of Health, Ministry of Health, Yerevan, Armenia; h Republican Specialized Scientific Practical Medical Center of Oncology and Radiology, Tashkent, Uzbekistan; i Institute of Oncology, Chisinau, Moldova; j P A Hertsen Moscow Oncology Research Center—Branch of FSBI National Medical Research Radiological Center of the Ministry of Health of the Russian Federation, Moscow, Russia; k Kazakh Institute of Oncology and Radiology, Almaty, Kazakhstan; l National Center for Disease Control and Public Health, Tbilisi, Georgia; m National Center of Oncology, Baku, Azerbaijan; n Scientific and Production Centre for Preventive Medicine of the Ministry of Health, Bishkek, Kyrgyzstan; o International Higher School of Medicine, IUK Academic Consortium, Bishkek, Kyrgyzstan; p NN Alexandrov National Cancer Centre of Belarus, Minsk, Belarus

## Abstract

**Background:**

Screening for breast cancer and cervical cancer in the newly independent states of the former Soviet Union is largely opportunistic, and countries in the region have among the highest cervical cancer incidence in the WHO European Region. We aimed to compare the stage-specific distributions and changes over time in breast cancer and cervical cancer incidence in the newly independent states of the former Soviet Union.

**Methods:**

We collected breast cancer and cervical cancer incidence data from official statistics from Armenia, Azerbaijan, Belarus, Georgia, Kazakhstan, Kyrgyzstan, Republic of Moldova, Russian Federation, Ukraine, and Uzbekistan for the years 2008–17 by tumour, node, metastasis (TNM) stage, and by age where population-based cancer registry data were available. We used log-linear regression to quantify the changes over time in age-standardised rates.

**Findings:**

During the period 2013–17, more than 50% of breast cancer cases across the analysed countries, and more than 75% of breast cancer cases in Belarus, Kazakhstan, and Ukraine, were registered at stages I–II. The proportion of stage I breast cancer cases was highest in the screening age group (50–69 years) compared with other ages in Moldova and the Russian registries, but was highest in those aged 15–49 years in Georgia and Ukraine. Breast cancer stage-specific incidence rates increased over time, most prominently for stage I cancers. For cervical cancer, the proportions of cancers diagnosed at a late stage (stages III and IV) were high, particularly in Moldova and Armenia (>50%). The proportion of stage I cervical cancer cases decreased with age in all countries, whereas the proportions of late stage cancers increased with age. Stage-specific incidence rates of cervical cancer generally increased over the period 2008–17.

**Interpretation:**

Our results suggest modest progress in early detection of breast cancer in the newly independent states of the former Soviet Union. The high proportions of early-stage disease in the absence of mammography screening (eg, in Belarus) provide a benchmark for what is achievable with rapid diagnosis. For cervical cancer, there is a need to tackle the high burden and unfavourable stage-specific changes over time in the region. A radical shift in national policies away from opportunistic screening toward organised, population-based, quality-assured human papillomavirus vaccination and screening programmes is urgently needed.

**Funding:**

Union for International Cancer Control, WHO Regional Office for Europe, and Ministry of Health of Ukraine.

## Introduction

The stage of cancer at diagnosis is a major determinant of cancer survival. In most countries, population-based data on stage are incomplete and lack comparability, even for the most common cancer sites. Only a small number of cancer registries, mostly in high-income settings, are able to determine complete tumour, node, metastasis (TNM) stage, whereas many instead use summary staging systems, which are not necessarily compatible with clinical practice.^1^ By contrast, in the newly independent states of the former Soviet Union, which have mostly retained their historical data collection systems dating back to the early 1950s, TNM stage is one of the variables that are mandatorily required in health-care statistics reporting, and is rarely missing in cancer registration forms and cancer registry reports.^2^ In addition to the cancer registration system relying on passive incidence data collection and active follow-up as described previously,^2^, ^3^ another remnant of the Semashko health-care system of the former Soviet Union is the dispensary system, opportunistic exams available annually at the primary health-care level (dispensaries).^4^

Breast and cervical cancers are major contributors to cancer morbidity and mortality and are subject to the large inequities observed in access to health-care services and interventions globally.^5^ Specifically, for cancers amenable to early detection, such as breast and cervical cancers, stage distribution reflects awareness of cancer symptoms, delays in seeking care, access to care, and the effectiveness of screening programmes for the target age groups.^6^

**Research in context**

**Evidence before this study**

Population-based data on cancer stage are vital for monitoring screening and early detection programmes and planning cancer control activities. In this study, we focused on the stage distributions and incidence trends of breast and cervical cancer given their high contribution to cancer burden and frequent inadequate screening practices in the newly independent states of the former Soviet Union. The information on cancer registration and screening practices collected during the activities in the scope of the partnership between IARC and the WHO Regional Office for Europe in supporting cancer surveillance was used to identify collaborators and support data interpretation. We searched PubMed using the terms “TNM stage”, “breast cancer”, “cervix cancer”, “incidence trends”, “Russia”, “Central Asia”, and “former Soviet Union” with no date restrictions. We used the membership lists of the International Association of Cancer Registries and the European Network of Cancer Registries to identify European cancer registry websites and search them for available data by TNM stage. We scrutinised the available site visit reports at IARC and WHO Regional Office for Europe for information on cervical and breast cancer screening practices in the newly independent states of the former Soviet Union. We assessed identified documents in English, Russian, and Czech languages.

**Added value of this study**

To our knowledge, this study is the first combined investigation of breast cancer and cervical cancer stage-specific distributions and changes over time in the newly independent states of the former Soviet Union. We observed later stages at diagnosis of breast cancer and cervical cancer in the newly independent states of the former Soviet Union compared with other European countries, as well as increasing stage-specific changes in incidence over time. Our results indicate that current breast cancer and cervical cancer screening practices in the newly independent states of the former Soviet Union are far from optimal, and provide a benchmark for breast and cervical cancer control planning in countries in the region.

**Implications of all the available evidence**

Screening practices for breast cancer and cervical cancer that are not evidence driven remain common in the newly independent states of the former Soviet Union. Transition to population-based, quality-assured human papillomavirus (HPV) screening, with the introduction of HPV vaccination, would catalyse progress towards cervical cancer elimination. For breast cancer, the decision whether to implement rapid diagnosis or organised population-based mammography screening programmes should be tailored based on national situation analyses. Advancing the quality of cancer registry data and strengthening the role of such data in cancer control will be critical for planning, monitoring, and evaluating progress.

Breast cancer incidence in the newly independent states of the former Soviet Union is generally lower than in European countries, with estimated age-standardised rates (world standard population) ranging from 19·5 cases per 100 000 population (Tajikistan) to 57·5 cases per 100 000 population (Georgia) in 2020, while mortality is similar or even higher (from 8·0 cases per 100 000 population in Tajikistan to 23·5 cases per 100 000 population in Georgia).^7^ The dispensary system, which includes regular clinical breast examinations for women of a broad age range, remains intensively practiced in some newly independent states of the former Soviet Union (eg, Belarus), but has been practically withdrawn in others, such as Georgia and Kyrgyzstan (WHO, unpublished). According to the WHO *Country Capacity Survey 2019*,^8^ most newly independent states of the former Soviet Union have mammography screening programmes, but overall coverage is low (mostly <50%), and screening is usually offered on an opportunistic basis with suboptimal quality assurance. The target age includes those aged 50–69 years in seven countries where mammography screening is available, but extends to women aged 40–49 years or younger in Georgia, Kazakhstan, Russian Federation, and Turkmenistan.^9^

Compared with other European countries, newly independent states of the former Soviet Union have high cervical cancer incidence (age-standardised rate >15·0 per 100 000 population in Kazakhstan and Kyrgyzstan, and 16·3 per 100 000 population in Moldova, compared with 7·0 per 100 000 population in western Europe, according to GLOBOCAN 2020 estimates).^7^ This high incidence is mainly attributed to insufficient organised screening programmes and inadequate diagnostic capacity.^9^ Although free-of-charge annual cervical screening is available for women of reproductive age, the quality of cytology is frequently poor. In the former Soviet Union, cervical smears were stained by Romanowski-Giemsa rather than Papanicolaou staining. Even though this practice is not evidence-based, it remains common in the region.

The aim of this study was to analyse stage-specific distributions and changes over time in breast cancer and cervical cancer incidence in the newly independent states of the former Soviet Union, to compare our results with European countries with available population-based data on TNM stage, and to interpret the collected data in the context of national cancer control activities in the region.

## Methods

### Study design

We defined newly independent states of the former Soviet Union as Armenia, Azerbaijan, Belarus, Georgia, Kazakhstan, Kyrgyzstan, Republic of Moldova, Russian Federation, Tajikistan, Turkmenistan, Ukraine, and Uzbekistan, excluding the Baltic countries (Estonia, Latvia, and Lithuania) that form part of the EU and as such abide by EU recommendations and guidelines on breast cancer and cervical cancer screening (a corresponding updated and evidence-based set of guidelines is not available for the newly independent states of the former Soviet Union). Contacts and collaborations with these countries have already been established in scope of the partnership between the International Agency for Research on Cancer (IARC) and the WHO Regional Office for Europe (WHO EURO) in supporting cancer surveillance in the newly independent states of the former Soviet Union. We contacted national cancer registries and cancer statistics offices in 12 newly independent states and identified collaborators, to whom we sent letters of invitation, data collection templates, and study protocols (in English and Russian).

We collected aggregated incidence data, defined using the International Classification of Diseases Tenth Revision (ICD-10)^10^ codes for breast cancer (C50) and cervical cancer (C53), as well as cervical carcinoma in situ (D06) by 5-year age group and calendar year, as well as the following corresponding data quality indicators: the proportion of microscopically verified cases, the proportion of patients registered posthumously (a specific indicator in the newly independent states of the former Soviet Union referring to cases registered based on autopsy or death certificate only^3^), and the mortality to incidence ratio, where available. For the mortality to incidence ratio, cancer mortality data were extracted from the WHO Mortality Database.^11^ No individual data were requested or used.

We received data from ten (83%) of 12 countries; Turkmenistan and Tajikistan did not submit data. Population-based data for Kazakhstan were provided by age group, but without stratification by stage. Information on cervical carcinoma in situ was available in four countries (Belarus, Georgia, Russia, and Ukraine).

### Data sources

Population counts or estimates for the period 2008–17 were received from the statistics office of each country. For Ukraine, population data were not available for the Crimea (2014–17) and Donetsk and Luhansk regions (2015–17). Thus, the data for these respective regions and periods were not included in the analysis.

We used official cancer statistics data from Armenia, Azerbaijan, Belarus, Georgia, Kazakhstan, Kyrgyzstan, Moldova, Russia, Ukraine, and Uzbekistan. The obligatory reporting of cancer statistics in the newly independent states of the Soviet Union involves collection and processing of predefined paper-based registration forms at the medical statistics office of a regional oncology centre. Reporting is required for all cases of malignant and in situ neoplasms (ICD-10 codes C00–C96 and D00–D09).^12^ In addition to the standard variables required for cancer registration, the registration form includes additional clinical information, such as TNM stage (for all solid tumours, including female genital cancers), details of treatment, and follow-up for disease progression. Stage distributions of cancers of visually accessible sites are reported as an indicator of preventive activities of regional oncology services.^12^ The data are reported from regional to national oncology centres, as well as annually to the Ministry of Health in an aggregated format. This data collection and reporting system has been preserved in most newly independent states, which continued the use of standard paper-based forms after the dissolution of the former Soviet Union.

The annual report to the Ministry of Health contains two tables with data on the number of new cases or patients by cancer site (ICD-10 codes) as well as TNM stage, with stages I and II grouped in most countries. Female and male breast cancers by stage are not reported separately.

Internationally published population-based cancer registry data from newly independent states of the former Soviet Union are sparse. Saint Petersburg (Russia) and Belarus cancer registries, included in *Cancer Incidence in Five Continents* volumes from 1983, continued submitting data every 5 years, with some additional registries from the region included in later volumes.^13^

We used population-based cancer registry data from Belarus, Georgia, Moldova, Ukraine (national), and Russia (60 regions combined). Additional requests for data were sent to the population-based cancer registries of Arkhangelsk and Samara (both included in *Cancer Incidence in Five Continents* volume XI), Tomsk, and the north western federal regions of Russia. We collected aggregate data by site, year, 5-year age groups, and TNM stage, excluding patients aged 0–14 years. Data on stage I and stage II cancers were available separately from the population-based cancer registry data.

### Data analysis

We chose to present the stage distributions for official statistics (overall) as well as for population-based data (by age group) for the time period 2013–17 to enable comparisons with other countries. To assess these data in the context of screening, we examined breast cancer cases by stage for the age groups 15–49 years, 50–69 years, and ≥70 years, and for cervical cancer for age groups 15–34 years, 35–59 years, and ≥60 years. Population-based cancer registry data for the complete 10-year period (2008–17) were available only for Ukraine, Belarus, and the Russian subnational registries. Truncated (age ≥15 years) age-standardised rates were calculated using the Segi-Doll world standard population.^14^ We fitted log-linear regression models to the truncated age-standardised rates to calculate the estimated annual percentage change in incidence by country and cancer site. Given well known difficulties in comparing staging data in different settings–eg, as a result of variations in the completeness and accuracy of the source information—we refrained from providing statistical measures of uncertainty. We used R (version 3.6.3) software for all analyses.

### Role of the funding source

The funder of the study had no role in study design, data collection, data analysis, data interpretation, or writing of the report. All authors had full access to all of the data and the final responsibility to submit for publication.

## Results

The description of data sources and data quality indicators is shown in the table. The proportion of patients diagnosed posthumously was <2% in all countries (data not shown).

**Table:** Description of data sources and data quality indicators for breast cancer (C50) and cervical cancer (C53) incidence in participating countries

	**Last census (year)**	**Oldest age group (years)**	**Official report, stages I–II combined**	**TNM edition**	**Proportion of microscopically verified cases**		**Mortality to incidence ratio (%; 2013–17)**		**Population-based cancer registry data used for analysis (years)**
					C50	C53	C50	C53	
Armenia	2011	≥85	Yes	No information	85·6%	97·7%	49·7% (2016)	23·8% (2016)	No
Azerbaijan	2009	≥75	Yes	No information	91·2%	93·5%	..	..	No
Belarus	2019	≥85	No	6 (2008–11); 7 (2012–17)	99·1%	99·7%	29·4% (2014)	38·2% (2014)	Yes (2008–17)
Georgia	2014	≥85	No	7	79·0%	78·5%	40·3% (2015)	65·2% (2015)	Yes (2015–17)
Kazakhstan	2009	≥85	No	6 (2008–11) 7 (2012–17)	95·8%	96·8%	31·6% (2015)	35·6% (2015)	No
Kyrgyzstan	2009	≥70	Yes	5	92·2%	95·7%	42·2% (2015)	45·3% (2015)	No
Moldova	2014	≥80	No	6 (2008–14) 7 (2015–17)	..	..	48·7% (2016)	51·1% (2016)	Yes (2016–17)
Russia	2010	≥85	No	5 (2008–13) 7 (2014–17)	97·3%	98·3%	35·5% (2015)	41·1% (2015)	Yes (2008–17)
Ukraine	2001	≥85	Yes	4 (2008–11) 6 (2012–17)	92·3%	98·6%	41·1% (2017)	39·1% (2017)	Yes (2008–17)
Uzbekistan	1989	≥65	Yes	6 (2008–15) 7 (2015–17)	97·7%	97·3%	45·9% (2016)	48·5% (2016)	No

**Figure 1:**
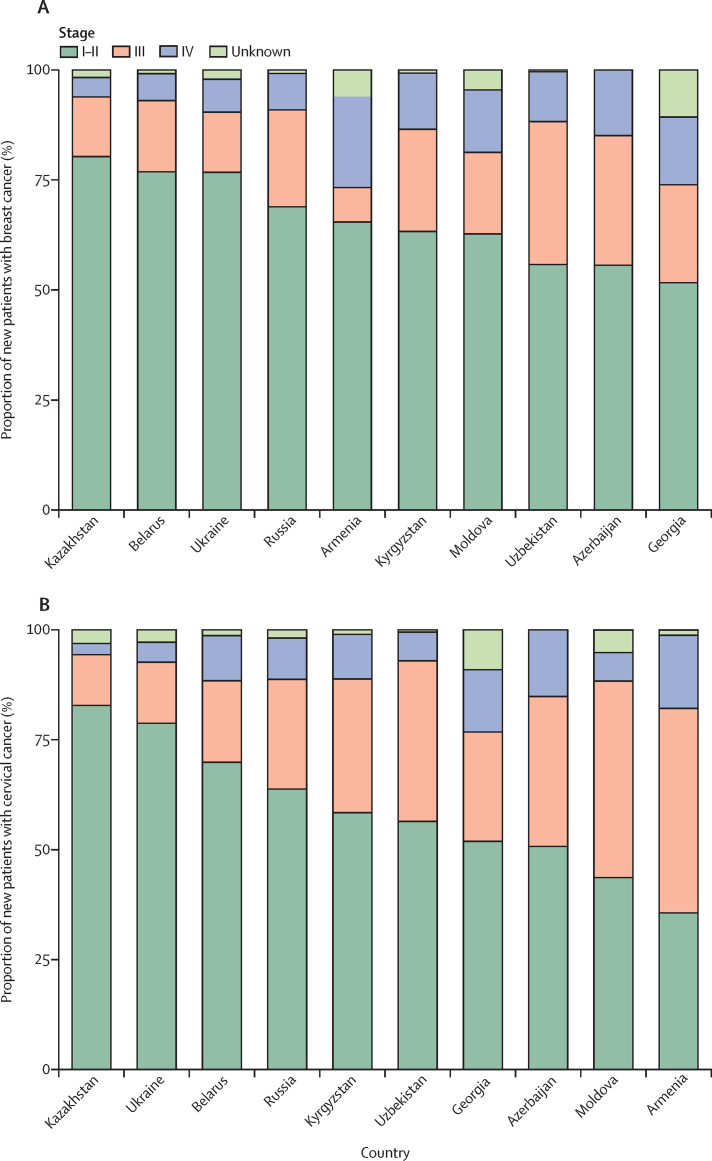
Proportion of new patients with breast cancer (A) and cervical cancer (B) or cases reported in official statistics by stage per diagnosis and country, 2013–17 Male breast cancer cases are included. Data show the number of patients, except for Belarus and Russia (number of cases). Data for Moldova are for 2013–16.

**Figure 2:**
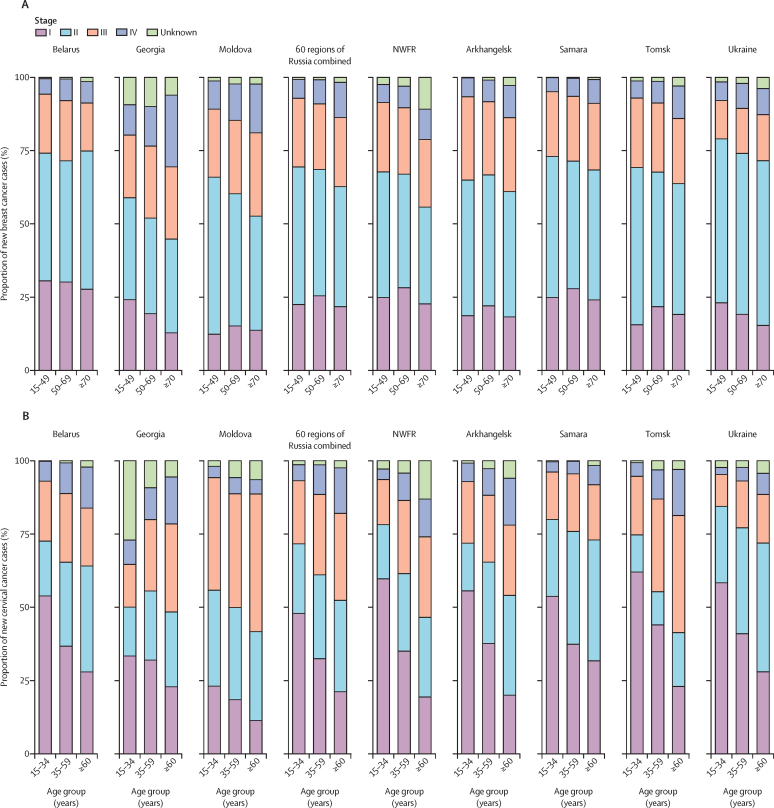
Proportion of new breast cancer (A) and cervical cancer (B) cases by stage and age group for each participating population-based cancer registry, 2013–17 Data for Georgia are for 2015–17. Data for Moldova are for 2016–17. NWFR=north western federal regions of Russia.

According to official statistics reports, over 50% of breast cancer cases in all countries were registered at stages I–II, whereas in Belarus, Kazakhstan, and Ukraine this proportion exceeded 75% (figure 1A; appendix p 1). More than 35% of breast cancer diagnoses were at late stages (III–IV) in Azerbaijan, Georgia, Kyrgyzstan, and Uzbekistan (figure 1A; appendix p 1). According to the population-based data, the proportion of stage I breast cancer cases was highest in the youngest age group in Belarus, Georgia, and Ukraine, but in Moldova and Russia most stage I cases were diagnosed in those aged 50–69 years (figure 2A). Georgia and Moldova had a large proportion of breast cancers diagnosed at stages III and IV, particularly in those aged 70 years and older.

For cervical cancer, at least 70% of cases were registered as stage I or II in Kazakhstan, Ukraine, and Belarus. By contrast, in Moldova and Armenia, the proportion of stage III-IV diagnoses was high (>50%; figure 1B; appendix p 1). According to the population-based data, the proportion of stage I cervical cancer cases decreased with age in all countries, whereas the proportions of cases diagnosed at a late stage (stages III and IV) increased with age, exceeding 50% in Moldova and the Tomsk region in those aged 60 years and older (figure 2B). Most invasive cervical cancers diagnosed at younger ages (15–34 years) were diagnosed at stage I, except in Moldova.

**Figure 3:**
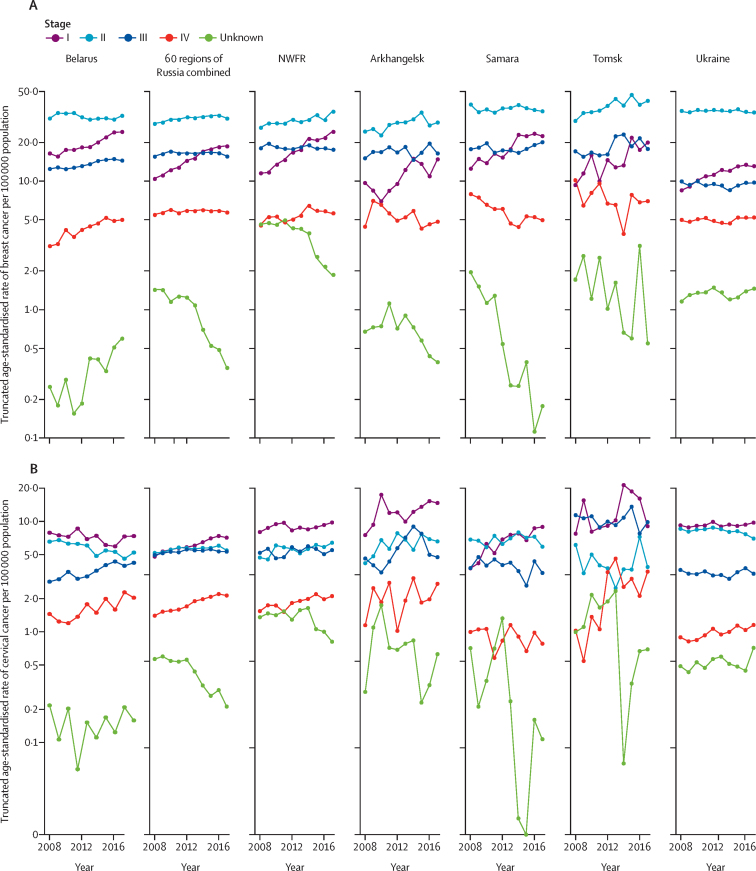
Changes over time in truncated age-standardised incidence of breast cancer (A) and cervical cancer (B) by stage and participating population-based cancer registry, 2008–17 NWFR=north western federal regions of Russia.

The number of cases and truncated (≥15 years) age-standardised rates by cancer site in the participating cancer registries 2013–17 alongside corresponding population data are available in the appendix (p 1). Truncated breast cancer incidence increased in all countries between 2008 and 2017 (appendix p 3). Analysis of stage-specific changes over time indicated large and uniform increases in stage I breast cancer incidence, with the estimated annual percentage change ranging from 5% to 9% (figure 3A; appendix p 2). Incidence of stage II breast cancer was the highest overall and was increasing in all regions other than Belarus, Ukraine, and Samara. Incidence of stage III breast cancer was stable or increasing across all regions, whereas the incidence of stage IV breast cancer increased in Belarus and the north western federal regions of Russia, but declined or was stable in the remaining regions. The incidence of cancers of unknown stage decreased in all Russian regions, but increased in Belarus and Ukraine.

No decreases in truncated cervical cancer rates for all stages combined were observed over the 2008–17 period (appendix p 3). Changes over time in stage-specific incidence of cervical cancer varied across countries (figure 3B; appendix p 2). In Belarus, the incidence of cervical carcinoma in situ was higher than for invasive cancer stages and increased from 2008 to 2017 (estimated annual percentage change 8%; appendix p 2), as did the incidence of stage III and stage IV disease, whereas the incidence of stage I and stage II disease decreased (figure 3B). In all other countries, the highest incidences were for stage I disease, increasing over time (figure 3B; appendix p 3). Incidence rates at late stages were mostly stable (stage III) or increasing (stage IV).

## Discussion

We examined the stage distributions at diagnosis of breast cancer and cervical cancer in the newly independent states of the former Soviet Union, two frequent cancers in the region, as markers of the general awareness of symptoms of these diseases, possible delays in the system in seeking and obtaining access to care, and the effectiveness of screening interventions within each country.^6^ Although our results suggest modest progress in breast cancer prevention in the newly independent states of the former Soviet Union, the extent of the burden and the unfavourable stage-specific incidence changes over time of cervical cancer continue to be of concern.

Breast cancer in the newly independent states of the former Soviet Union is most frequently diagnosed at earlier stages (I–II), although the proportions of late stage breast cancers remain high—with more than 35% of diagnoses at stages III–IV in Azerbaijan, Georgia, Kyrgyzstan, and Uzbekistan—particularly compared with European countries (eg, England, UK, 13% stages III–IV in 2014; Czech Republic 17·3% in 2014–16).^15^, ^16^ The proportion of stage IV breast cancer exceeded 11% in six of ten newly independent states of the former Soviet Union, whereas in the above mentioned European countries they were 5% and 6·6%, respectively.^15^, ^16^ In the newly independent states with available population-based registry data, breast cancer was most commonly diagnosed at TNM stage II, which contrasts with data available from Norway, the UK, Czech Republic, and Belgium, where stage I is most commonly reported.^15^, ^16^, ^17^, ^18^

The uniformly increasing incidence over time of stage I breast cancers observed in Belarus, several Russian regions, and Ukraine, as well as larger proportions of stage I cancers in those aged 50–69 years compared with other age groups in Moldova and Russian regions suggests a possible positive effect of opportunistic mammography and clinical breast examination screening, or increased awareness of symptoms and rapid diagnosis within this age group. Similar increases in the incidence of localised disease, alongside stable diagnoses of metastatic breast cancer, have been reported from other countries during the implementation phase of organised screening, with a subsequent flattening of early-stage cancer incidence rates after full implementation of the programme.^17^, ^19^

In this study, we observed generally stable incidence of late-stage breast cancer cases. Increasing incidence of early-stage cancers with concomitant stable trends at metastatic stages is suggestive of overdiagnosis, which is a common concern with mammography screening.^20^ However, late-stage cancers could be biologically distinct, being more aggressive and more likely to present as interval cancers.^21^ Despite modest screening coverage, overdiagnosis cannot be ruled out as a contributor to the marked increases in stage I cancers that were observed across the newly independent states of the former Soviet Union. The absence of a decline in late-stage cancers (apart from in selected Russian regions) is consistent with overall increases in breast cancer incidence in the newly independent states.

The newly independent states of the former Soviet Union have among the highest incidence of cervical cancer in the WHO European Region, particularly among central Asian countries, where cervical cancer incidence ranks second in frequency after breast cancer.^22^ Our results indicate that, in contrast with recorded rates in most regions of the world, cervical cancer incidence is increasing in the newly independent states of the former Soviet Union. Stage-specific results show the high proportion of cervical cancers diagnosed at late stages (III–IV), particularly in Moldova and Armenia, compared with corresponding proportions of 20·3% in Norway, 21% in Northern Ireland (UK), and 36·6% in Czech Republic.^16^, ^23^, ^24^ The absence of a decline in the incidence of late-stage cervical cancers in Belarus, Russian regions, and Ukraine suggests little progress in prevention over the last decade, despite the potential of effective screening to prevent more than 150 000 cervical cancer cases by 2040 in Russia alone.^9^ Screening practices in the region are still largely untargeted and opportunistic, based predominantly on Romanowsky-Giemsa staining techniques, and without standard guidelines for management of abnormal cytology findings.^25^ Although use of Romanowsky-Giemsa staining is common, there are considerable intercountry and intracountry variations across the newly independent states of the former Soviet Union, with the choice of staining technique depending on the hospital or laboratory, the regulations in place, and resources available. WHO EURO are working towards compiling a document repository on screening practices in the newly independent states. As each newly independent state of the former Soviet Union is in a different phase of health-care system transition, accurate diagnosis and access to treatment for all cancers remains a challenge in many countries, given the lack of availability of chemotherapy or radiotherapy in some countries and a reliance on out-of-pocket payments for health services. In some countries, barriers to accessing diagnoses are higher than access to treatment, substantially impacting cancer stage at diagnosis. Additionally, suboptimal pathology practices, exemplified by the persisting use of Romanowski-Giemsa staining for cervical cancer screening, can further delay accurate diagnosis, staging, and treatment.

A major strength of this study is the use of previously unpublished data on TNM stage that are available from cancer registration systems across the newly independent states of the former Soviet Union.^1^ Yet the parallel use of different TNM editions for registration and treatment purposes, often due to the absence of consensus within the clinical community, is a potential limitation of the results presented here. Another limitation concerns the data quality of the cancer registries in the newly independent states, particularly in relation to adherence to international guidelines. Only six of 12 newly independent states of the former Soviet Union (excluding Baltic states) have national or subnational population-based cancer registries in operation, with incidence data from Belarus, Ukraine, and four regional registries from Russia considered high quality given their inclusion in the most recent volume of *Cancer Incidence in Five Continents*.^26^ Variations in the reported mortality to incidence ratio could reflect differences in survival, as well as in completeness and validity of source data, and thus this indicator is difficult to interpret given the varying quality of mortality data in the region.^27^ In Georgia and Moldova, population-based cancer registries were established less than 5 years ago, and it is too early for the analysis of changes over time. We were unable to include data for Kazakhstan in the analysis of changes over time by stage, as this information was missing. Elsewhere in the newly independent states of the former Soviet Union, despite existing cancer reporting systems, the implementation of population-based cancer registries remains in an early phase. Nonetheless, even with the challenge of ensuring comparability of data across populations, we believe that the data on TNM stage at diagnosis collected from all cancer hospitals in the newly independent states—via the long-established cancer reporting systems in each country—are representative of the distributions in the respective populations, and are therefore comparable.

To prevent premature deaths, certain breast cancer and cervical cancer practices, such as opportunistic screening across non-targeted age groups with non-standard techniques (eg, Romanowsky-Giemsa testing for cervical cancer, and ultrasound for screening of breast cancer among young women), need to be replaced by evidence-based and resource-adequate interventions. Results of the WHO Cervical Cancer Modelling Consortium tasked with informing the WHO Cervical Cancer Elimination Strategy have shown that girls-only human papillomavirus (HPV) vaccination with 90% coverage alongside twice-lifetime HPV-based screening could halve cervical cancer incidence in low-income and middle-income countries by 2048.^28^ Alongside the introduction of HPV vaccination, moving from current opportunistic screening practices to population-based, quality-assured HPV screening for women beyond vaccination age would place the newly independent states of the former Soviet Union on the road to cervical cancer elimination as a public health problem over the coming decades.

With respect to breast cancer, the decision over whether to implement rapid diagnosis or organised population-based mammography screening programmes should be tailored based on a situation analysis of each country, including the assessment of capacity for mammography screening and follow-up of positive cases, but also the public health capacity to establish, maintain, and manage quality assurance, including centralised screening databases that encompass all of the required functions. WHO EURO has issued guidance on screening (in four languages, including Russian) for policymakers to better understand how to increase the effectiveness of screening, to maximise benefit, and minimise harm.^29^ In the absence of adequate infrastructure and strong health systems, rapid diagnosis is a preferred approach to achieve a shift towards earlier stage distributions of diagnosed cancer cases, leading to improved survival.^29^ From this perspective, the high proportions of early-stage disease in the absence of mammography screening (eg, in Belarus) provide a benchmark for what could be achievable via rapid diagnosis.

In conclusion, strengthening the role that registries play in cancer control and improving the quality of data in the newly independent states of the former Soviet Union will be crucial for monitoring progress in prevention and treatment in the region. With this vision, the IARC-led Global Initiative for Cancer Registry Development Program and WHO EURO are working in partnership to support robust cancer surveillance data to inform national polices across the region.

## Data sharing

The data that support the findings of this study are available from the first author upon reasonable request and with the permission of the contributing cancer registries.
