# Supplementary material for: Comparison of breast cancer and cervical cancer stage distributions in ten newly independent states of the former Soviet Union: a population-based study
Source: Lancet Oncol. 2021 Mar;22(3):361–9. doi: 10.1016/S1470-2045(20)30674-4 (PMC8014987; doi:10.1016/S1470-2045(20)30674-4)
Supplement: Supplementary appendix [file mmc1.pdf]

THE LANCET
Oncology

## Supplementary appendix

This appendix formed part of the original submission and has been peer reviewed. We post it as supplied by the authors.

Supplement to: Ryzhov A, Corbex M, Piñeros M, et al. Comparison of breast cancer and cervical cancer stage distributions in ten newly independent states of the former Soviet Union: a population-based study. *Lancet Oncol* 2021; **22:** 361–69.

## Web Appendix (Supplementary material)

# Comparisons of breast and cervical cancer stage distributions in ten newly independent states of the former Soviet Union: a population-based study

Anton Ryzhov, Marilys Corbex, Marion Piñeros, Anton Barchuk, Diana Andreasyan, Sayde Djanklich, Vadim Ghervas, Olga Gretsova, Dilyara Kaidarova, Konstantin Kazanjan, Fuad Mardanli, Yuriy Michailovich, Elena Ten, Alesya Yaumenenka, Freddie Bray, Ariana Znaor

**Section of Cancer Surveillance, International Agency for Research on Cancer, Lyon, France** (A Ryzhov PhD, M Piñeros MD, F Bray PhD, A Znaor MD);
**National Cancer Registry of Ukraine, National Cancer Institute, Kyiv, Ukraine** (A Ryzhov, Y Michailovich PhD);
**Taras Shevchenko National University of Kyiv, Ukraine** (A Ryzhov);
**World Health Organization Regional Office for Europe, Copenhagen, Denmark** (M Corbex PhD);
**Tampere University, Faculty of Social Sciences/Health Sciences, Tampere, Finland** (A Barchuk PhD);
**NN Petrov National Research Medical Center of Oncology, Saint-Petersburg, Russian Federation** (A Barchuk);
**National Institute of Health, Ministry of Health, Yerevan, Armenia** (D Andreasyan PhD);
**Republican Specialized Scientific-Practical Medical Center of Oncology and Radiology, Tashkent, Uzbekistan** (S Djanklich MSc);
**Institute of Oncology, Chisinau, Republic of Moldova** (V Ghervas MD);
**P. A. Hertsen Moscow Oncology Research Center – branch of FSBI National Medical Research Radiological Center of the Ministry of Health of the Russian Federation, Moscow, Russian Federation** (O Gretsova PhD);
**Kazakh Institute of Oncology and Radiology, Almaty, Kazakhstan** (D Kaidarova PhD);
**National Center for Disease Control and Public Health, Tbilisi, Georgia** (K Kazanjan MSc);
**National Center of Oncology, Baku, Azerbaijan** (F Mardanli PhD);
**Scientific and Production Centre for Preventive medicine of the Ministry of Health, Bishkek, Kyrgyzstan** (E Ten PhD);
**International Higher school of medicine, IUK academic consortium, Bishkek, Kyrgyzstan** (E Ten);
**and N.N. Alexandrov National Cancer Centre of Belarus, Minsk, Belarus** (A Yaumenenka PhD)

**Supplementary Table 1. Average annual female population, number of cases and truncated age-standardized rates (TASR) by cancer site in the participating cancer registries, 2013-2017.**

| Country / Region | Population | | Female breast | | | Cervix | | | Cervix *in situ* | |
|---|---|---|---|---|---|---|---|---|---|---|
| | Average annual n | % 15+ | n | TASR | GLOBOCAN-2020 | n | TASR | GLOBOCAN-2020 | n | TASR |
| Belarus | 5,072,524 | 85·3 | 4,312 | 72·1 | 75·4 | 916 | 18·0 | 16·5 | 1,441 | 35·3 |
| Georgia (2015-17) | 1,938,342 | 82·4 | 1,793 | 87·7 | 83·3 | 338 | 18·2 | 15·4 | 37 | 2·4 |
| Moldova (2016-17) | 1,843,730 | 85·1 | 846 | 44·3 | 61·7 | 300 | 16·9 | 23·6 | - | - |
| Russia, 60 regions | 62,130,628 | 85·2 | 53,223 | 72·2 | 79·6 | 12,484 | 20·4 | 20·5 | 2,518 | 4·9 |
| Ukraine | 20,389,448 | 86·1 | 15,035 | 63·0 | 63·9 | 4,399 | 22·1 | 20·7 | 1,100 | 6·7 |
| Total | 91,374,672 | | 75,209 | | | 18,437 | | | 5,096 | |

Abbreviations: TASR – truncated age-standardized rates; GLOBOCAN-2020[1] – truncated age-standardized rates in 2020 estimated by the IARC

**Supplementary Table 2. Average annual female population, number of cases and truncated age-standardized rates (TASR) by cancer site in participating Russian subnational cancer registries, 2013-2017.**

| Country / Region | Population | | Female breast | | Cervix | | Cervix *in situ* | |
|---|---|---|---|---|---|---|---|---|
| | Total | % 15+ | n | TASR | n | TASR | n | TASR |
| Russia, NWFR | 6,518,134 | 86·2 | 6,337 | 79·3 | 1,516 | 23·6 | 288 | 5·5 |
| Russia, Arkhangelsk | 629,472 | 84·2 | 499 | 65·7 | 152 | 29·4 | 72 | 14·7 |
| Russia, Samara | 1,739,966 | 86·0 | 1,761 | 81·9 | 346 | 19·7 | 84 | 5·4 |
| Russia, Tomsk | 569,044 | 84·2 | 530 | 87·3 | 179 | 33·5 | 14 | 2·8 |

Abbreviations: TASR – truncated age-standardized rates; NWFR – North-Western Federal Region

**Supplementary Table 3. Number and proportion of new breast and cervix cancer patients/cases reported in official statistics by stage at diagnosis and country, 2013-2017.**

| Country | Stage I-II | | Stage III | | Stage IV | | Stage unknown | | Total | |
|---|---|---|---|---|---|---|---|---|---|---|
| | n | % | n | % | n | % | n | % | n | % |
| Breast (C50) | | | | | | | | | | |
| Armenia | 3,327 | 65·4 | 398 | 7·8 | 1,056 | 20·8 | 304 | 6·0 | 5,085 | 100·0 |
| Azerbaijan | 4,731 | 55·6 | 2,512 | 29·5 | 1,266 | 14·9 | 0 | 0·0 | 8,509 | 100·0 |
| Belarus | 16,286 | 76·8 | 3,450 | 16·3 | 1,300 | 6·1 | 164 | 0·8 | 21,200 | 100·0 |
| Georgia | 3,850 | 51·6 | 1,664 | 22·3 | 1,160 | 15·5 | 791 | 10·6 | 7,465 | 100·0 |
| Kazakhstan | 17,217 | 80·3 | 2,919 | 13·6 | 943 | 4·4 | 369 | 1·7 | 21,448 | 100·0 |
| Kyrgyzstan | 1,868 | 63·3 | 684 | 23·2 | 376 | 12·8 | 21 | 0·7 | 2,949 | 100·0 |
| Moldova | 2,576 | 62·7 | 760 | 18·5 | 584 | 14·2 | 186 | 4·5 | 4,106 | 100·0 |
| Russia | 227,550 | 68·8 | 72,974 | 22·1 | 27,272 | 8·3 | 2,733 | 0·8 | 330,529 | 100·0 |
| Ukraine | 53,006 | 76·7 | 9,499 | 13·7 | 5,108 | 7·4 | 1,496 | 2·2 | 69,109 | 100·0 |
| Uzbekistan | 7,987 | 55·8 | 4,676 | 32·6 | 1,611 | 11·2 | 52 | 0·4 | 14,326 | 100·0 |
| Cervix (C53) | | | | | | | | | | |
| Armenia | 438 | 35·6 | 573 | 46·5 | 205 | 16·7 | 15 | 1·2 | 1,231 | 100·0 |
| Azerbaijan | 939 | 50·7 | 632 | 34·1 | 282 | 15·2 | 0 | 0·0 | 1,853 | 100·0 |
| Belarus | 3,116 | 69·9 | 824 | 18·5 | 461 | 10·3 | 59 | 1·3 | 4,460 | 100·0 |
| Georgia | 706 | 51·8 | 340 | 24·9 | 194 | 14·2 | 123 | 9·0 | 1,363 | 100·0 |
| Kazakhstan | 7,297 | 82·8 | 1,015 | 11·5 | 223 | 2·5 | 277 | 3·1 | 8,812 | 100·0 |
| Kyrgyzstan | 1,431 | 58·4 | 744 | 30·4 | 249 | 10·2 | 25 | 1·0 | 2,449 | 100·0 |
| Moldova | 539 | 43·7 | 551 | 44·7 | 79 | 6·4 | 65 | 5·3 | 1,234 | 100·0 |
| Russia | 52,137 | 63·8 | 20,387 | 24·9 | 7,636 | 9·3 | 1,575 | 1·9 | 81,735 | 100·0 |
| Ukraine | 16,167 | 78·7 | 2,861 | 13·9 | 922 | 4·5 | 585 | 2·8 | 20,535 | 100·0 |
| Uzbekistan | 4,066 | 56·4 | 2,629 | 36·5 | 472 | 6·6 | 37 | 0·5 | 7,204 | 100·0 |

**Supplementary Table 4. Truncated age-standardized rates (TASR) and estimated annual percentage change (EAPC) for selected registries, breast cancer, 2008-2017.**

| Country / Region | Stage I | | | | Stage II | | | | Stage III | | | | Stage IV | | | | Stage Unknown | | | |
|---|---|---|---|---|---|---|---|---|---|---|---|---|---|---|---|---|---|---|---|---|
| | **2008** | **2017** | **EAPC,%** | **p** | **2008** | **2017** | **EAPC** | **p** | **2008** | **2017** | **EAPC** | **p** | **2008** | **2017** | **EAPC** | **p** | **2008** | **2017** | **EAPC** | **p** |
| Belarus | 16·4 | 24·2 | **5** | <0·0001 | 30·7 | 32·2 | -1 | 0·1978 | 12·4 | 14·5 | **2** | 0·0001 | 3·1 | 5·0 | **6** | 0·0001 | 0·3 | 0·6 | **13** | 0·0083 |
| Russia, 60 regions | 10·5 | 18·8 | **7** | <0·0001 | 28·2 | 31·0 | **1** | 0·0021 | 15·7 | 15·7 | 0 | 0·9195 | 5·5 | 5·7 | 0 | 0·1907 | 1·4 | 0·4 | **-15** | 0·0001 |
| Russia, NWFR | 11·5 | 24·2 | **9** | <0·0001 | 26·0 | 34·7 | **2** | 0·0008 | 18·0 | 17·6 | 0 | 0·2534 | 4·5 | 5·6 | **3** | 0·0156 | 4·6 | 1·9 | **-10** | 0·0009 |
| Russia, Arkhangelsk | 9·7 | 14·8 | **7** | 0·0094 | 24·3 | 28·6 | **3** | 0·0293 | 15·1 | 16·4 | 1 | 0·5 | 4·4 | 4·8 | -2 | 0·1894 | 0·7 | 0·4 | **-6** | 0·0474 |
| Russia, Samara | 12·5 | 22·4 | **8** | 0·0001 | 39·5 | 35·0 | 0 | 0·7551 | 17·7 | 20·1 | 1 | 0·4676 | 7·9 | 5·0 | **-5** | 0·0024 | 1·9 | 0·2 | **-27** | 0·0001 |
| Russia, Tomsk | 9·3 | 20·0 | **8** | 0·0071 | 29·5 | 42·2 | **4** | 0·0028 | 17·1 | 17·8 | 3 | 0·0786 | 10·2 | 7·0 | -4 | 0·2414 | 1·7 | 0·5 | -9 | 0·1795 |
| Ukraine | 8·4 | 13·0 | **5** | <0·0001 | 35·0 | 34·1 | 0 | 0·637 | 9·9 | 9·7 | 0 | 0·516 | 5·0 | 5·2 | 0 | 0·356 | 1·2 | 1·4 | 1 | 0·285 |

Abbreviations: EAPC – estimated annual percentage change; NWFR – North-Western Federal Region

Note: If the slope coefficient of the log-liner model is significantly different from a zero gradient at p=0.05, we consider the EAPC be statistically significant (marked bold).

**Supplementary Table 5. Truncated age-standardized rates (TASR) and estimated annual percentage change (EAPC) for selected registries, cervical cancer, 2008-2017.**

| Country / Region | In situ | | | | Stage I | | | | Stage II | | | | Stage III | | | | Stage IV | | | | Stage Unknown | | | |
|---|---|---|---|---|---|---|---|---|---|---|---|---|---|---|---|---|---|---|---|---|---|---|---|---|
| | **2008** | **2017** | **EAPC,%** | **p** | **2008** | **2017** | **EAPC,%** | **p** | **2008** | **2017** | **EAPC,%** | **p** | **2008** | **2017** | **EAPC,%** | **p** | **2008** | **2017** | **EAPC,%** | **p** | **2008** | **2017** | **EAPC,%** | **p** |
| Belarus | 20·0 | 42·7 | **8** | <0·0001 | 7·9 | 7·4 | -2 | 0·1795 | 6·6 | 5·2 | **-4** | 0·0012 | 2·9 | 4·2 | **5** | 0·0004 | 1·5 | 2·0 | **6** | 0·0042 | 0·2 | 0·2 | 1 | 0·7725 |
| Russia, 60 regions | 2·7 | 5·8 | **9** | <0·0001 | 4·8 | 7·1 | **5** | <0·0001 | 5·2 | 5·5 | 1 | 0·0522 | 5·0 | 5·3 | 1 | 0·0555 | 1·4 | 2·1 | **5** | <0·0001 | 0·6 | 0·2 | **-11** | 0·0001 |
| Russia, NWFR | 6·8 | 5·9 | -3 | 0·1431 | 8·0 | 9·8 | 1 | 0·2272 | 4·7 | 6·4 | **3** | 0·0215 | 5·2 | 5·5 | 1 | 0·434 | 1·5 | 2·1 | **4** | 0·0017 | 1·4 | 0·8 | **-5** | 0·0346 |
| Russia, Arkhangelsk | 4·0 | 17·2 | **17** | 0·0001 | 7·5 | 14·7 | 5 | 0·0723 | 4·2 | 6·6 | **4** | 0·0481 | 4·6 | 4·7 | 5 | 0·1502 | 1·1 | 2·7 | 4 | 0·3161 | 0·3 | 0·6 | -6 | 0·3712 |
| Russia, Samara | 1·0 | 7·5 | **22** | <0·0001 | 3·8 | 8·9 | **9** | 0·0003 | 6·9 | 5·9 | 0 | 0·7732 | 3·8 | 3·4 | -3 | 0·1842 | 1·0 | 0·8 | -2 | 0·4377 | 0·7 | 0·1 | - | - |
| Russia, Tomsk | 2·3 | 3·6 | 3 | 0·6086 | 7·7 | 9·0 | 5 | 0·2308 | 6·1 | 3·9 | -1 | 0·8657 | 11·4 | 9·9 | -1 | 0·5051 | 1·0 | 3·5 | **18** | 0·0153 | 1·0 | 0·7 | -14 | 0·2142 |
| Ukraine | 6·1 | 6·9 | **2** | 0·0008 | 9·2 | 9·7 | 1 | 0·197 | 8·6 | 7·0 | **-2** | 0·0235 | 3·6 | 3·4 | 0 | 0·8980 | 0·9 | 1·2 | **3** | 0·0014 | 0·5 | 0·7 | 2 | 0·221 |

Abbreviations: EAPC, estimated annual percentage change; NWFR – North-Western Federal Region

Note: If the slope coefficient of the log-liner model is significantly different from a zero gradient at p=0.05, we consider the EAPC be statistically significant (marked bold).

**Supplementary Figure 1. Trends in truncated age-standardised cancer incidence rates (TASR) of breast (A) and cervix (B) by participating PBCRs, 2008-2017**

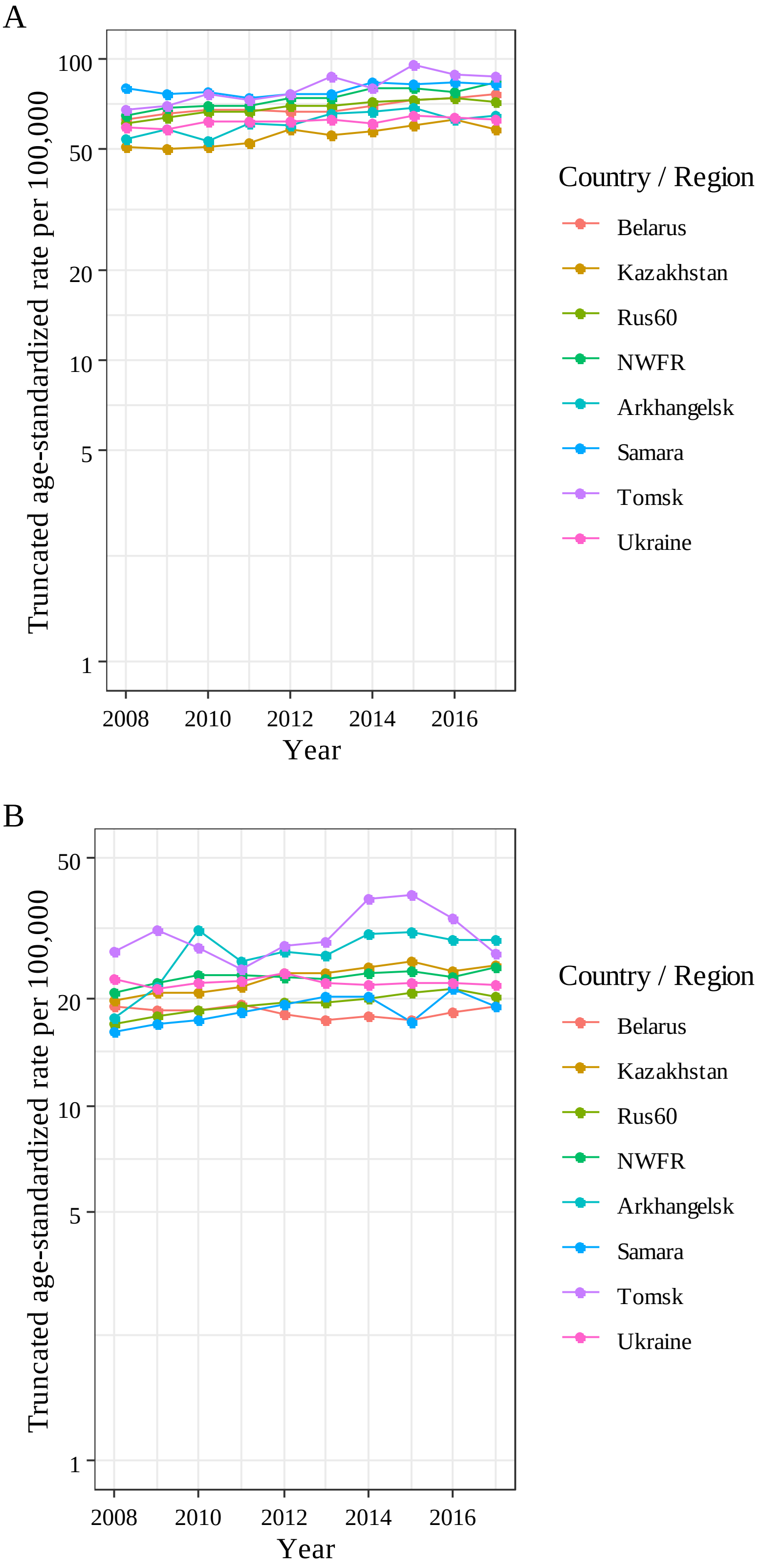


Legend: TASR – truncated age-standardized rates (World Standard Population); PBCR – population-based cancer registry; Rus60 – 60 regions of Russia combined; NWFR – North-Western Federal Region

**References**

1 Sung H, Ferlay J, Siegel RL, Laversanne M, Soerjomataram I, Jemal A, Bray F. Global Cancer Statistics 2020: GLOBOCAN estimates of incidence and mortality worldwide for 36 cancers in 185 countries. *CA Cancer J Clin*, in press.
